# SLC5A8 Gene, A Transporter of Butyrate: A Gut Flora Metabolite, Is Frequently Methylated in African American Colon Adenomas

**DOI:** 10.1371/journal.pone.0020216

**Published:** 2011-06-08

**Authors:** Hassan Brim, Krishan Kumar, Javad Nazarian, Yetrib Hathout, Amir Jafarian, Edward Lee, William Green, Duane Smoot, Jong Park, Mehdi Nouraie, Hassan Ashktorab

**Affiliations:** 1 Department of Medicine and Cancer Center, Department of Pathology, Howard University, College of Medicine, Washington, D.C., United States of America; 2 Research Center for Genetic Medicine, Children's National Medical Center, Washington, D.C., United States of America; 3 Division of Cancer Prevention and Control, H. Lee Moffitt Cancer Center, Tampa, Florida, United States of America; Vanderbilt University Medical Center, United States of America

## Abstract

**Background:**

Colon cancer is one of the leading causes of cancer related deaths. Its impact on African Americans (AAs) is higher than in the general population both in the incidence and mortality from the disease. Colon cancer aggressiveness in AAs as well as non-frequent check-ups and follow up in this population have been proposed as ways to explain the observed discrepancies. These facts made the detection of early carcinogenesis markers in this population a priority.

**Materials and Methods:**

Here, we analyzed 50 colon adenomas from AA patients for both microsatellite instability (MSI) and the methylation status of SLC5A8 gene. This gene's product is involved in the transport of butyrate that has anti-proliferative properties through its effects on histone acetylation and gene expression. A proteomic analysis to check the expressed histones in adenoma and normal tissues was also performed.

**Results:**

The analyzed samples displayed 82% (n = 41) methylation level of SLC5A8 gene in adenomas. The MSI-H (high) adenoma were about 18% (n = 9) while the rest were mostly MSS (microsatellite stable) with few MSI-L (Low). No association was found between SLC5A8 methylation and the MSI status. Also, there was no association between SLC5A8 methylation and the sex and age of the patients. However, there were more right sided adenomas with SLC5A8 methylation than the left sided ones. The proteomic analysis revealed distinct histone expression profiles between normal and adenoma tissues.

**Conclusion:**

SLC5A8 is highly methylated in AA colon adenomas which points to its potential use as a marker for early detection. The MSI rate is similar to that found in colon cancer tumors in AAs. These findings suggest that both processes stem from the same epigenetic and genetic events occurring at an early stage in colon carcinogenesis in AAs.

## Introduction

Colon cancer is the third most common form of cancer and the second leading cause of cancer-related deaths in American adults. The incidence of this disease in African Americans (AA) is higher than in the general population [1], [2], [3]. African Americans are also more likely to die from colon cancer than others. There are different participating factors in colon carcinogenesis. Genetic factors impact gene or genome structures and integrity while epigenetic parameters have an effect on DNA structure and expression [4], [5], [6] while environmental factors such as diet and its interaction with the gut flora impacts the colon mucosa through the metabolic products' effects on colon tissue homeostasis. The metabolic byproducts are channeled through the colon mucosa by a set of genes of which the primary function is molecules' transport. The SLC gene family encodes a group of proteins that are involved in such transport through cell membranes. Mutations or lack of expression of these genes have been implicated in many disorders [7]. SLC5A8, also called SMCT for sodium-coupled monocarboxylate transporter, is a member of the SLC gene family and have been extensively studied. This gene is expressed in the retina, brain, kidney, thyroid and in the gastrointestinal system [8], [9], [10], [11], [12], [13].

It has been shown that the SLC5A8 protein sit within the apical membrane of the intestinal tract where it is involved in the absorption of short chain fatty acids (e.g. butyrate) into the colon [10], [11]. Butyrate, a byproduct of bacterial fermentation, has been proven to have anti-proliferative characteristics. As such, SLC5A8 gene product that is involved in its transport through the colon mucosa have been labeled as a tumor suppressor gene [11], [12].

It has also been shown that butyrate impacts cell proliferation through its effect on histones acetylation status. Histone modification is very instrumental in the expression level of genes within the cell. Consequently, butyrate transport by SLC5A8 gene product impacts the expression level of many genes that are likely involved in the negative control, anti-proliferative, of the cell cycle within the colon mucosa [12].

This gene's involvement in cancer has already been highlighted in many studies. Indeed, Park et al [10] have already shown that both pancreatic and prostate cancers display an SLC5A8 methylation level of 70% unlike in the adjacent non-tumor tissues [11].

A similar situation was reported by Bennett et al. in head and neck squamous cell carcinoma patients [8]. Using a Restriction Landmark Genomic Scanning technique, they found five methylation markers among which SLC5A8 gene. The other genes were SEPT9, FUSSEL18, EBF3 and IRX1 [8]. While studying posttranslational histones modifications in mixed lineage leukemia (MLL), Whitman et al., (2008) found that such modifications are generally associated with a hypermethylation profile in which SLC5A8 gene plays a central role [13].

A recent study by Thangaraju et al (2008) have shown that SLC5A8 is silenced in humans, in cell lines and in a mouse model of colon cancer [12]. Their findings strengthen earlier results by Li et al [9]. They also proved that the re-expression of SLC5A8 in colon cancer cell lines in the presence of butyrate leads to apoptosis through the up-regulation of pro-apoptotic genes and down-regulation of proliferative genes [12].

Most of the studies on SLC5A8 were done on cancer samples and none has checked in adenomas. As such, we here propose to analyze the methylation status of SLC5A8 gene in African American colon adenomas with the goal of finding early colon cancer markers in this group of patients to mitigate the high incidence of the disease in this group. Histones expression profiles was assessed using a proteomic analysis. Microsatellite instability, also an early event, was studied and data from both analysis were compared.

## Materials and Methods

### Ethics Statement

This study was approved by the Howard University Institutional Review Board (HUIRB), and the study exempt by HUIRB from consent form since we used archival samples and the patients could not be identified.

### Study population, and colon adenomas samples

Fifty archived and fresh frozen adenomas from 2003 to 2005 from Howard University were included in this study. All samples were evaluated and subjected to histological diagnosis by expert pathologists. Tissues were collected (with approval from above site's Institutional Review Boards) and clinical data was obtained (including race, age, and site of adenomas) with no identifiers. The adenoma and normal areas were diagnosed by a pathologist (EL, WG) using the H&E matching slides.

### DNA isolation and MSI analysis

The slides were microdissected to pinpoint the adenoma areas as well as normal areas from at least two slides. A pinpoint DNA extraction kit was used as recommended by the manufacturers (Zymo Research). DNA was extracted from fresh frozen adenoma samples using a Gentra DNA extraction kit (Gentra). The extracted DNA was used as template in PCR reactions with five microsatellite markers as previously described [5]. Briefly, markers for (BAT25, BAT26, NR21, NR22 and NR24) were used to evaluate MSI status of the analysed adenomas. PCR products were analyzed in a 3130 ABI GeneScan. The samples displaying DNA instability at only one of the markers were labeled MSI-L, those displaying instability with two or more markers were labeled MSI-H, and those displaying no instability with any of the five markers tested were labeled MSS.

### Sodium bisulfite treatment

DNA (200 to 500 ng) was subjected to a sodium bisulfite treatment to convert unmethylated cytosine to thymidine. A ZymoResearch DNA Easy Methylation kit was used according to the manufacturers' instructions. In brief, The DNA is incubated at 50° C for 16 hours in the presence of the conversion reagent, and then transferred to a column where it is being desulphonated for 15 min before its elution in 10 µl of elution buffer.

### Methylation-specific MS-PCR

**Figure 1:**
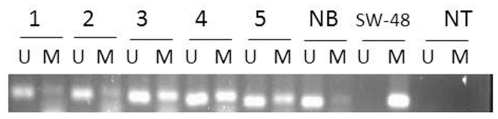
MSP analysis of the promoter region of SLC5A8 in five patients with adenoma. The presence of visible PCR product in lanes “U” indicates the presence of unmethylated genes of SLC5A8, the presence of product in lanes “M” indicates the presence of methylated genes. Water controls for PCR reaction are also shown as NT: no template. MSP of SLC5A8 in NB: Normal Blood lymphocyte DNA used as control for unmethylated at SLC5A8, SW48: colon cancer cell line used as a positive control. Patients # 3–5 are methylated, and patients # 1–2 are semimethylated.

The converted DNA was used as template in PCR reactions using two sets of primers: primers that selectively amplify methylated DNA: SLC5A8-M-F: 5′-TCGAACGTATTTCGAGGC and SLC5A8-M-R: 5′-ACAACGAATCGATTTTCCG [9]. The second set of primers to amplify unmethylated DNA had the following sequences: SLC5A8-U-F: 5′-TTGAATGTATTTTGAGGTG and SLC5A8-U-R: 5′-TCAATTTTCCAAAATCCC (Invitrogen, Carlsbad, CA) [9]. PCR reactions were performed using the primer pairs described above in the following reaction mix: 10X PCR buffer [16.6 mM ammonium sulfate, 67 mM Tris (pH 8.8), 6.7 mM MgCl2, and 10 mM 2-mercaptoethanol], dNTPs (each at 1.25 mM), primers (50 pmol each per reaction), and bisulfite-modified DNA (50 ng) in a final volume of 50 µl. Reactions were hot-started at 95°C for 5 min, after which 1.25 units of *Taq* polymerase (Life Technologies, Inc.) were added. PCR parameters for both sets of primers were: 95°C for 15 min followed by 40 cycles of 95°C for 30 s, 56°C for 45 s, 72°C for 45 s, and a final step of 72°C for 10 min then 4°C to cool. Controls for methylated and unmethylated DNA were DNA from the SW48 colon cancer cell line (ATCC, VA) and normal lymphocytes, respectively. Each PCR reaction product (10 µl) was directly loaded into a 2% agarose gel, which was later stained with ethidium bromide to allow DNA visualization under UV illumination. The presence of a band with primers specific for unmethylated DNA and its absence with those specific for methylated DNA qualified the analyzed adenoma as unmethylated. However, when a band for methylated DNA was present for the analyzed adenoma, we defined the sample as methylated (Figure 1) (or semi-methylated if both unmethylated and methylated bands were displayed on gel).

### Immunohistochemistry

**Figure 2:**
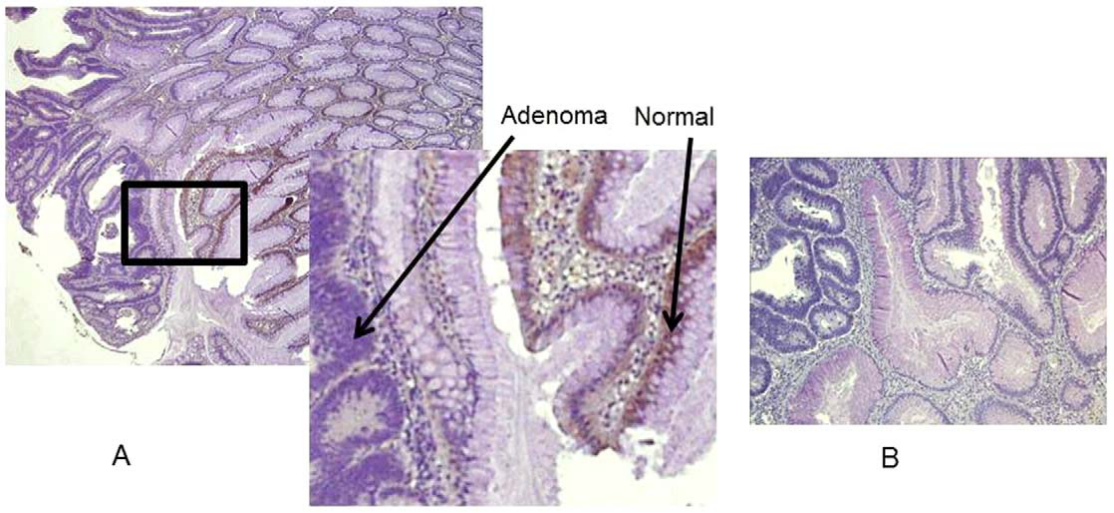
SLC5A8 expression in human colon normal and adenoma. Characteristic cytoplasmic staining of normal colon, and adenoma, was determined by immunohistochemistry, using antibodies against SLC5A8 (A), and negative control without primary antibody (B). Original magnifications, ×4 magnification, except for enlargements (insets), which are ×40.

Tissue sections on positively charged slides were prepared from formalin-fixed, paraffin-embedded specimens Four-micron thick sections were cut by microtome (Leica Microsystems, Inc., Bannockburn, IL) and transferred to adhesive-coated slides. The slides were de-paraffinized by heating at 55°C for 30 min and by three 5-min washes with xylene. Tissues were rehydrated by a series of 5-min washes in 100%, 95%, and 80% ethanol and distilled water. Antigen retrieval was accomplished by heating the tissues at 95°C for 12 min in 10 mmol/L sodium citrate (pH 6.0). Tissue were immunostained using the avidin-biotin-peroxidase method. After blocking with universal blocking serum (DAKO Diagnostic, Mississauga, Ontario, Canada) for 30 min, tissues were incubated with a polyclonal anti-SLC5A8 antibody (provided by as a gift by V. Ganapathy). at 4°C overnight. Then sections were incubated with biotin-labeled secondary antibody and streptavidin-peroxidase for 30 minutes each (DAKO Diagnostic). Tissues were developed with a 3, 3′-diaminobenzidine substrate (Vector Laboratories, Burlington, Ontario, Canada) and counterstained with hematoxylin. Negative controls were created by omitting the anti-SLC5A8 antibody during primary antibody incubation (Figure 2).

Two pathologists (EL, WG) blinded to tissue origins scored stained tissue sections for intensity of SLC5A8 immunostain and percent of cells stained. Intensity of stain was ranked subjectively on a scale of 0 to 3, where “0” represents the absence of staining and “3” is maximal staining. The percent of cells stained was estimated on a scale of 0 to 3, with 0 for 0%, 1 for 1–33%, 2 for 34–66%, and 3 for greater than 66% of cells stained. SLC5A8 expression score for each core section was calculated as the product of immunostain intensity and the percent of cells stained.

### Proteomic analysis

**Figure 3:**
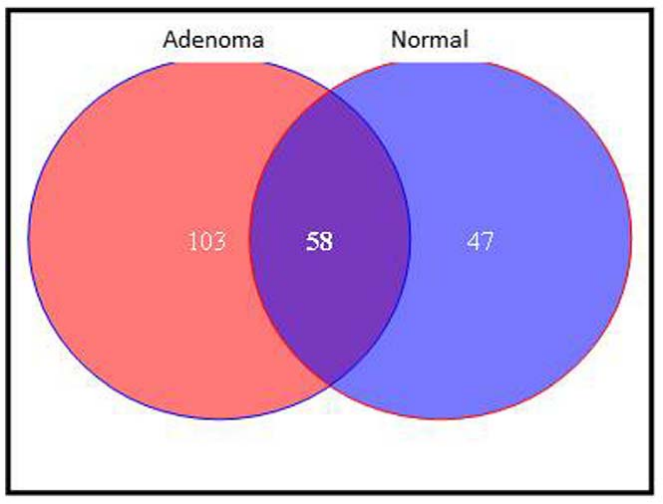
Adenoma and normal areas were extracted from microdissected of FFPE samples. Extracted peptides from adenoma and normal regions were separately subjected to LC-MS-MS/MS. Data were searched against human database and analyzed by Visualize software. After stringent filtration, 161 proteins were found to be expressed by the adenoma and 105 by the normal region . Of these, 103 proteins were found to be exclusively expressed in the adenoma.

Adenoma and normal areas were extracted as described before [14] from sectioned FFPE samples. Extracted peptides from adenoma and normal regions were separately subjected to Liquid Chromatography-Mass Spectrometry-Mass Spectrometry/Mass Spectrometry (LC-MS-MS/MS). Data were searched against human database and analyzed by Visualize software. After stringent filtration, 161 proteins were found to be expressed by the adenoma and 105 by the normal region (Figure 3). Of these, 103 proteins were found to be exclusively expressed by the adenoma (Table 1). Furthermore, we analyzed the protein expression levels between the normal and the adenoma. Of the 58 proteins that were expressed in both normal and adenoma, 51 proteins showed either <1.5 or >1.5 fold change in adenoma when compared to the control sample (Table 2).

**Table 1:** SLC5A8 methylation and clinicopathological properties colon adenoma.

	SLC5A8 unmethylated	SLC5A8 methylated	P value
Mean (SD) age	58 (3.0)	63 (2.2)	0.29
Gender			0.48
Female	3 (14%)	19 (86%)	
Male	6 (21%)	22 (79%)	
Colon Location of samples			0.09
Right colon	2 (%)	21 (91%)	
Left Colon	5 (29%)	12 (71%)	
MSI status			0.72
MSI-L	7 (17%)	34 (83%)	
MSI-H	2 (22%)	7 (78%)	

**Table 2:** SLC5A8 methylation and its relation with the colon adenoma subtype and adenocarcinomas.

	SLC5A8 unmethylated (%)	SLC5A8 methylated (%)	P value
TA	4 (17)	20 (83)	0.73
TVA	2 (14)	12 (86)	
VA	1 (50)	1 (50)	
Adenoca.	1 (20)	4 (80)	
Polyp	1 (20)	4 (80)	

### Statistical analysis

To establish correlations between SLC5A8 methylation and other parameters, different statistical analysis were performed. For categorical variables, such as MSI status, patient gender and adenoma location, we used a chi-square test. For SLC5A8 methylation association with patient age, we used t test. For SLC5A8 methylation vs. type of adenomas, the Fisher exact test was used.

## Results

### Characteristics of patient samples

Of the 50 patient samples included in this study, 28 (56%) were male and 22 (44%) were females (Table 1). The mean age in this group of patients was 62.5 years. The mean age in both male and females was similar, 62.14 for female patients and 62.78 for male patients. Twenty three (46%) of the samples were located on the right side of the colon while 17 (34%) were specifically left sided (Table 1). For ten patients (20%), the samples were from both sides of the colon. Twenty four (48%) of the samples were tubular adenomas, 14 (28%) were tubulovillous adenomas, 2 (4%) were villous adenomas, 5 (10%) were adenocarcinomas and 5 (10%) were polyps. For the adenocarcinoma samples, DNA was extracted from the adenomas sites on the slides.

### Microsatellite instability

DNA from the 50 samples was analyzed for microsatellite instability, a phenotype that is the result of mutation or epigenetic silencing of DNA mismatch repair genes such as MLH1 genes. The samples were classified into three categories depending on how many tested markers showed instability with MSI-H: 2 or more markers, MSI-L: one marker and MSS: stable for all markers (Table 1). Within the 50 samples, 9 (18%) displayed instability at 2 or more markers and as such are MSI-H, 10 (20%) were MSI-L and 31 (62%) were MSS. Five MSI-H (55.5%) were right sided while 2 (22.2%) were left sided, another 2 (22.2%) were located on both sides of the colon. For the non-MSI-H samples, 19 (46.3%) were right sided while 15 (36.6%) were left sided, another 7 (17%) were on both sides of the colon.

### SLC5A8 methylation

The methylation specific PCR analysis of the 50 samples using SLC5A8 specific primers revealed that 82% (41) samples were methylated at this gene level (Figure 1 & Table 1).

### SLC5A8 methylation and patient gender

A statistical analysis using the Pearson chi-square test has revealed that SLC5A8 methylation distributes equally among the two genders. Nineteen female patients and 22 male patients displayed methylation. The p-value (0.477) showed that there were no statistically significant difference between the two genders (Table 1).

### SLC5A8 methylation and patient age

The mean age for patients with unmethylated SLC5A8 was 58.22 (Std. Dev. 9.03) while the patients with methylated SLC5A8 gene had an average age of 63.43 (Std. Dev. 13.78). Using a t test, the difference between the two groups was not found to be statistically significant with a p- value of 0.2851 (Table 1).

### SLC5A8 methylation and tumor location

Forty samples that were clearly defined as right or left sided were included in this analysis using the Pearson chi2 method. Twenty one (91.30%) right sided samples were methylated for SLC5A8 while only 2 (8.7% were unmethylated. Twelve left sided (70.59%) samples were methylated while 5 (29.41%) were unmethylated. A p-value of 0.088 reflects a tendency for SLC5A8 to associate more with the right colon samples than the left ones (Table 1).

### SLC5A8 methylation and the MSI status

For this analysis, MSI-L and MSS samples were grouped together as non MSI-H adenomas. Two (22%) MSI-H samples were unmethylated while 7 (78%) were methylated. Within the non MSI-H group , 7 (17%) samples were unmethylated while 34 (82.93%) were methylated. A p-value of 0.716 reflected that there was no difference of statistical significance between the two groups and as such the data don't support any association between the MSI status and SLC5A8 methylation (Table 1).

### SLC5A8 methylation and diagnosis

SLC5A8 methylation profile was similar in all 5 categories of samples used (TA, TVA, VA, AdeCA, and polyps) except for villous adenomas for which we had only two samples. The overall methylation rate was around 80% which is similar to the methylation rate obtained in all 50 samples combined (Table 2).

### Immunohistochemistry

We performed immunohistochemistry on several adenomas using an antibody that is specific to SLC5A8 protein (provided by as a gift by V. Ganapathy). In the analysed specimens, all adenomas showed no staining with the antibody while normal tissue did (Figure 2). Adenomas cytoplasmic staining were scored 3 (greater than 66% of cells stained). These results further confirm the methylation data and their effect on the gene's expression.

### Proteomics

A proteomic analysis from colon adenomas and matched normal has been performed. The extracted peptides were run through an LC-MS-MS/MS. Data were searched against human database and analyzed by Visualize software. After stringent filtration, 161 proteins were found to be expressed by the adenoma and 105 by the normal region (Figure 3). Of these, 103 proteins were found to be exclusively expressed by the adenoma. Furthermore, we analyzed the protein expression levels between the normal and the adenoma. Of the 58 proteins that were expressed in both normal and adenoma, 51 proteins showed either <1.5 or >1.5 fold change in tumor when compared to the control sample. Of note SLC5A8 transported compound, butyrate affects chromatin structure through its effect on histones. The proteomic data revealed that there are some histones that are specifically expressed in adenomas (H2A type 2a and Histone 2B type 1) while others were at least two folds more expressed in adenoma compared to normal (H2A type3, type1, type1-C, type 1-E, histone H3.3 and H3.1) that were all 2.64 folds more in adenomas. Histones H2A type 1-F and H2A/z were 3.19 folds more in adenoma while Histones H2A type4 and histone 4 were 4.09 and4.27 folds more in adenoma than normal respectively.

## Discussion

Earlier studies have checked the methylation status of SLC5A8 gene in many cancerous tissue and cell lines: prostate, head and neck, pancreas, colon and blood. All of these studies have pointed to a high methylation level of this gene among others [8], [9], [10], [13], [15]. SLC genes are known to be involved in the transport of many solutes that differ from one gene to another and for the same gene from one organ to the other [7] SLC5A8 gene is involved in the transport of butyrate, propionate, and pyruvate, all of which are inhibitors of histone deacetylases [16]. SLC5A8 acts on colon tissue homeostasis mainly through its transport of butyrate, a byproduct of gut flora metabolism. Indeed, in colon cancer cell lines that were demethylated by 5-aza-cytidine, an up-regulation of pro-apoptotic and down-regulation of anti-apoptotic genes was noticed only if butyrate was supplemented in the medium [12].

In this study, we established the methylation status of SLC5A8 in colon adenomas in African Americans, a population at high risk of developing colon cancer. Our finding that 82% of samples were methylated strengthen earlier findings in cancerous samples and point to the early occurrence of this methylation in the carcinogenic process. Our recent CpG islands microarray analysis of different types of adenomas further confirmed our findings [17]. In these microarray studies, SLC5A8 gene came out always methylated. The MSI analysis have displayed an 18% MSI-H rate that is similar to the one obtained with a study of 222 AA colon carcinomas that showed 19.8% MSI-H [6]. As such both microsatellite instability and SLC5A8 methylation occur very early in colon tumorigenesis. However, since not all tumors are initiated as a result of DNA MMR defects in expression or sequence, microsatellite instability phenotype would only define a small subset of tumors (∼20%). The co-occurrence of SLC5A8 methylation and the MSI phenotype in our samples might also point to the fact that both result from the same methylation event since the MSI phenotype is generally caused by an epigenetic silencing of DNA mismatch repair genes. Within the MSI-H group, 22% were not methylated for SLC5A8, also 83% SLC5A8 methylated samples were non-MSI-H. These data reflects that there is some level of specificity during the DNA methylation process. Indeed, our statistical analysis has clearly shown that there was no association between SLC5A8 methylation and the MSI phenotype. DNA methylation is known to be part of the aging process. Our data with SLCA8 methylation seems to be non-age specific. Many of the patients in this study were not very old and the difference in age between the groups SLC5A8 methylated vs. unmethylated was not statistically significant. Consequently the high level methylation observed in this study is likely linked to the tumorigenic process. It is noteworthy that the mean age of our group of patients (62.5 years) is in agreement with the data obtained with our large scale study of 222 colon carcinoma in AAs [6]. There was 44% of patients in this study who were over 60 years while Kumar et al (2008) reported 64.9% of AA colon cancer patients over that age. Sex-wise, SLC5A8 methylation was evenly distributed between male and female patients. It has already been reported [18] that certain loci are differentially methylated between the two genders. Such is not the case for SLC5A8, at least within the colon tissue where its function does not seem to have any gender specific particularity. There was a noticeable trend to have SLC5A8 methylation linked to samples that are right sided. Ninety one percent of right sided samples vs. 70.59% left sided were methylated. Proximal colon is known to be the site of more methylation events than the distal one [15], [19]. SLC5A8 methylation distributed equally among the 5 different categories of samples used in this study. Tubulovillous, tubular, adenocarcinoma and polyps all showed a similar level of SLC5A8 methylation (∼80%) that was comparable to the value with the entire 50 samples analyzed here. Villous adenomas (n = 2) showed 50% methylation, a rate mainly attributed to the low number of samples from this category. Consequently, the methylation level seems not to be associated with any specific kind of adenomas in our group of African American patients. Dong et al., have pointed to a major role of SLC5A8 methylation in the early stages leading to serrated lesions [20] while Goldstein et al., has reported that SLC5A8 methylation along with BRAF mutation are major actors in the initiation of sessile serrated adenomas[21]. The recently named serrated adenoma correspond to adenomas with combined architectural features of hyperplastic polyps and classical adenoma. Most of the samples in this study are archived samples that have been classified prior to the advent of sessile adenomas nomenclature. It would not be surprising to find that some might fall in this group. However and since BRAF mutation seems to associate with SLC5A8 methylation in this group of adenomas, it would be unlikely. Indeed, BRAF mutation in African American colon cancer samples based on our previous study of 222 samples was found only in 7 cases out of 222 [6].

It is noteworthy to remember that butyrate, that is being transported by SLC5A8 gene product, has a direct impact on histones acetylation status. Ueno et al, have shown that in gastric cancer samples, histone H3 acetylation correlated directly with SLC5A8 expression and inversely with DNA methylation [22]. Our laboratory has already established that there was a direct correlation, that was statistically significant , between HDAC2 expression and colon cancer development in AAs [23]. Also, H3K12 and H4K18 acetylation status was decreased from normal to cancer AA samples although not in a statistically significant manner. These findings put together piece some elements of the puzzle of AA colon carcinogenesis where SLC5A8 and HDAC2 expression play major roles through their combined effects on gene expression.

The proteomic data also revealed a higher expression of many histones 2A of different types in adenomas vs. normal tissues. Histones 2A are known to affect chromatin structure and thus gene expression [24]. Li and Jun reported that butyrate induces profound changes in the expression of at least 450 genes in bovine kidney epithelial cells [25]. Such a tremendous effect would only be possible through chromatin remodeling where histones are involved.

The high level of SLC5A8 methylation in our study also fits the general picture about genes' methylation in AA colon cancer. A recent study from our laboratory revealed that AAs, when compared to Iranian patients, have higher levels of methylation at 13 candidate cancer genes [26], [27]. This methylation, like SLC5A8 methylation, was age and sex independent highlighting an important role of hypermethylation in AA colon carcinogenesis. Our exclusive choice to work with adenomas, and our inclusion of 5 polyps in this study strengthen that assumption and qualify SLC5A8 gene as a potential marker for early detection in this high risk group of patients.
